# A Green Triboelectric Nano-Generator Composite of Degradable Cellulose, Piezoelectric Polymers of PVDF/PA_6,_ and Nanoparticles of BaTiO_3_

**DOI:** 10.3390/s20020506

**Published:** 2020-01-16

**Authors:** Zhuangzhi Sun, Lu Yang, Sicheng Liu, Jintao Zhao, Zhiwei Hu, Wenlong Song

**Affiliations:** 1 Province Key Laboratory of Forestry Intelligent Equipment Engineering, College of Mechanical and Electrical Engineering, Northeast Forestry University, Harbin 150000, China; yanglu1995x@163.com (L.Y.); liusicheng@nefu.edu.cn (S.L.); 2 Key Laboratory of Bio-based Material Science & Technology, Ministry of Education, Northeast Forestry University, Harbin 150000, China; 3 Harbin University of Science and Technology, Rongcheng 264200, China; zhaojintao@hrbust.edu.cn

**Keywords:** nano-generator, cellulose, piezoelectric polymers, nanoparticles, performance

## Abstract

In this paper, a kind of green triboelectric nano-generator based on natural degradable cellulose is proposed. Different kinds of regenerated cellulose composite layers are prepared by a blending doping method, and then assembled with poly(tetrafluoroethylene) (PTFE) thin films to form tribioelectric nanogenerator (TENG). The results show that the open circuit output voltage and the short circuit output current using a pure cellulose membrane is 7.925 V and 1.095 μA. After adding a certain amount of polyamide (PA6)/polyvinylidene fluoride (PVDF)/barium titanate (BaTiO_3_), the open circuit output voltage peak and the peak short circuit output current increases by 254.43% (to 20.155 V) and 548.04% (to 6.001 μA). The surface morphology, elemental composition and functional group of different cellulose layers are characterized by Scanning Electronic Microscopy (SEM), Fourier transform infrared spectroscopy (FT-IR), X-ray diffraction (XRD), and tested by the electrochemical analyze. Moreover, after multiple assembly and rectification processing, the electrical output performance shows that the peak value of open-circuit output voltage and the peak value of short circuit output current increases by 132.06% and 116.13%. Within 500 s of the charge-discharge test, the single peak charge reached 3.114 V, and the two peak charges reached 3.840 V. The results demonstrate that the nano-generator based on cellulose showed good stability and reliability, and the application and development of natural biomaterials represented by cellulose are greatly promoted in miniature electronic sensing area.

## 1. Introduction

In nature, there are different kinds of energy, where mechanical energy has the characteristics of high energy density, diverse expressions and wide distribution, which is the preferred choice for energy harvesting and conversion. In previous studies, it has been proved that nano-generators can effectively convert mechanical energy into electrical energy [1,2,3,4], and the conversion efficiency depends on the degree of coupling between friction effect and electrostatic induction. Moreover, nano-generators have the characteristics of high output voltage, small size, light weight, low cost and good safety [5,6,7,8,9]. They are widely used in the fields of mobile electronic devices, sensing systems and biomedicine [10,11,12,13]. Now, it becomes the research focus in energy collection and conversion. 

Nowadays, there are more and more types of nano-generators and more mature energy harvesting devices are being developed, which mainly include piezoelectric [14,15,16,17,18,19,20], friction [21,22,23,24,25,26,27,28,29], electromagnetic [30,31,32] and other nano-generators, which can convert and store the collected energy or provide power for mobile electronic devices after a certain period of time. With the rise of the concept of green environmental protection, there is an urgent need to develop a green, environment-friendly and bio-degradable nano-generator. In 2016, the first biodegradable TENG was made from a synthetic polymer, polylactic acid-glycolic acid (PLGA), 3-hydroxybutyrate 3-hydroxyvalerate (PHBV) and polycaprolactone (PCL). Ding [33] et al. designed a novel solar-driven regenerative electrochemical system for simultaneous photoelectric energy harvesting and storage. With rational screening of redox species and comprehensive electrochemical study, a high Seebeck coefficient of −1.8 mV K^−1^ is achieved by solely exploiting earth-abundant materials based on the thermogalvanic effect. In 2019, the effect of piezoelectricity, internal resistance, and surface treatment on output is explained experimentally and theoretically, which illustrates that the extent of charge transfer has a strong connection with the piezoelectricity, internal resistance, and surface treatment of the composite [34]. However, these polymers are often expensive and contain certain harmful chemicals. Natural bio-materials which are compared with these synthetic polymers usually have low cost, wide distribution, easy processing, good biocompatibility, degradability and good film-forming properties. Simultaneously, they are appropriate for the construction of TENG [35,36,37,38], which has extensive application prospect in the fields of biomedicine, electronic sensing, etc.

In this study we propose a kind of environmentally friendly triboelectric nano-generator based on natural degradable cellulose, which is compared to some developed polymer-generators. At present, chitosan and cellulose are abundant in many natural vegetation sources and organisms. By utilizing the dissolution and regeneration characteristics of cellulose and ionic liquids, the regenerated cellulose film can be obtained as a friction layer, which can generate electricity by extrusion and friction, and test output electrical performance. At this foundation, PA6/PVDF with different electronegativity, and BaTiO_3_ with high dielectric constant and low dielectric loss, are added for performance optimization. For theoretically explain the effect of piezoelectric and internal resistance on the output, different types of triboelectric layer films are carried out test of electrochemical performance and characterization. The effect of piezoelectricity, internal resistance on output is explained theoretically by electrochemical performance, which illustrates that the extent of charge transfer has a strong connection with the piezoelectricity, internal resistance of the composite. In addition, the electrical output performance is tested after rectifying multiple assemblies. Then, the capacitor is powered for testing its charging and discharging performance within 1000 s. After that, power is supplied to microcontroller, and then drives SFM-27 buzzer and LCD screen. The nano-generator device proposed in this paper has good stability and reliability, and can be widely used in the field of energy harvesting, which greatly promotes the development of natural biomaterials in TENG and other miniature electronic sensor devices.

## 2. Experimental Preparation and Operating Principle

### 2.1. Experimental Materials

α-Cellulose (99.5) was purchased from Aladdin Chemical Company (Shanghai, China). The ionic liquid 1-butyl-3-methylimidazolium chloride ([Bmim]Cl, molecular weight 174.67 kDa, melting point 70 °C) was purchased from the Physical Chemistry Institute of Lanzhou (Lanzhou, China). Barium titanate (BaTiO_3_) (content not less than 99%, molecular weight 233.19 kDa), polyamide powder (PA6, (C_6_H_11_NO)_n_), polyvinylidene fluoride (PVDF, content not less than 99.5%, and molecular weight 825000) were purchased from Macleans Company (Harbin, China). Some commonly used chemical reagents (distilled water, etc.) were purchased from Yongchang Reagent Co. Ltd. (Harbin, China).

### 2.2. The Preparation of Different Types of Composite Cellulose Layers

[Bmim]Cl (5 g) is added to q beaker, and then stirred at 85 °C for 10 min. Then, α-cellulose (0.5 g) weighed by using an analytical balance, is added into the [Bmim]Cl, and the mixture is stirred at a low speed for 60 min at a temperature of 85 °C. A certain amount of PA6, PVDF and BaTiO_3_ are added and stirred at a low speed for 30 min at a temperature of 85 °C to prepare different kinds of cellulose substrate friction layers. Under room temperature and humidity conditions, the prepared friction layer solution is coated on a certain size of glass plate and stands for 1 h. After that, the membrane is placed in distilled water for 20 min. Each of the friction layer films, which named of Cel, Cel + PA6, Cel + PA6 + BaTiO_3_, Cel + PVDF, Cel + PVDF + BaTiO_3_, is removed on a glass plate.

**Figure 1:**
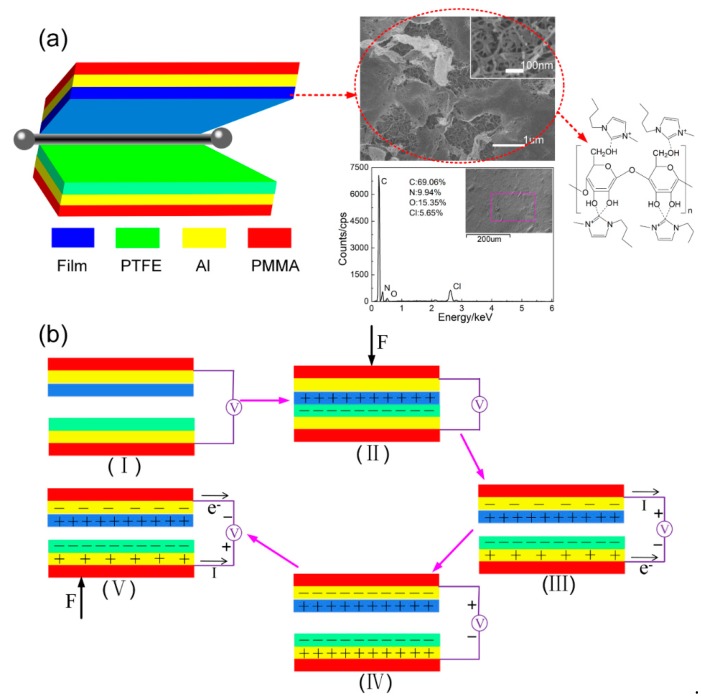
(**a**) Schematic diagram of cellulose composited nano-generator, (**b**) working principle diagram of cellulose composited nano-generator.

The nano-generator device based on degradable cellulose consists of six layers of top electrode (Al_1_), different cellulose layer, PTFE layer, bottom electrode (Al_2_). Due to the good mechanical properties of PMMA materials, it is used as the upper and lower encapsulation layers. The specific structure is shown in Figure 1a above. The electron microscope image of cellulose film with upper friction layer is shown on the right. Under high magnification, it can be seen that the surface of cellulose film is full of pore structure, which is convenient for ion transmission and also conducive to the attachment of granular PA6, PVDF and BaTiO_3_. EDS analyze results show that the presence of C, N and O elements proves the main composition of cellulose, and the presence of a small amount of Cl element is caused by incomplete phase exchange. Finally, the molecular formula of cellulose film is shown in Figure 1a.

### 2.3. Operating Principle

In this paper a nano-generator based on the piezoelectric contact and electrostatic induction is proposed. The plate area is 30 mm × 30 mm, the external force is 10 N, and the frequency is 1 Hz. The upper and lower friction layers contact or rub each other, and the inner surfaces of the two friction layers are charged with the same amount of different charges as shown in Figure 1b (the specific working mechanism see the supporting information). 

Different kinds of regenerated cellulose layers are prepared by blending doping method, and assembled with PTFE to form TENG. After rectification treatment, the capacitor is supplied with power to test its charge and discharge performance within 1000 s. After that, power is supplied to microcontroller, which is used to drive the alarm of a SFM-27 buzzer and light up the LCD screen (see Figure 1). 

## 3. Performance Testing and Analysis

### 3.1. FT-IR/XRD Characterization Analysis

**Figure 2:**
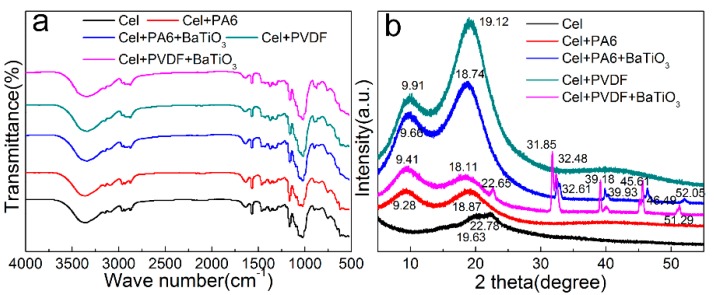
Characterization graphics of different types of friction layers: (**a**) FT-IR diagram, (**b**) XRD diagram.

By comparing the FT-IR patterns of different types of friction layers (Figure 2a), it can be seen that each material has similar functional group peak positions, FT-IR graph shows a similar trend. However, the sharpness of each peak is different, because C-H, C-F and N-F bonds are introduced before doping. This causes a change in the content, which is manifested by a change in the sharpness of the corresponding peak position and a certain shift in the peak position.

Figure 2b shows the XRD scan of different types of friction layers. After adding PA6, PVDF and BaTiO_3_, the XRD pattern of the diffraction peak angle of each friction layer was changed. Specifically, after dissolution and regeneration, the friction layer of pure cellulose shows a diffraction peak position at 19.63° and 22.78°, which is a typical cellulose II type. After the addition of PA6 and PVDF, a new diffraction peak appears at the positions of 9.28° and 9.91°. After the addition of BaTiO_3_, new diffraction peaks appear at around 32.61°, 39.93°, 46.49°, and 52.05°. In addition, the sharpness of the diffraction peak and the change of the peak width are caused by an increase in the crystallinity of the friction layer after the addition of the new substance, which is manifested by an increase in the intensity of the diffraction peak. The diffraction peaks of each friction layer are shifted rightward or leftward. This is because during the stirring process, the ions are replaced during the phase exchange, resulting in a change in the intermolecular and hydrogen bonding forces along with the change of the lattice size and it is represented by the diffraction peak shift. After adding different substances, the XRD diffraction peak sharpness change, peak shift and new peak position appear to change the internal structure and physical properties of the friction layer, which affect the output performance of the nano-generator. 

### 3.2. CV (Cyclic Voltammetry) Test Analysis

The electrochemical testing of the cellulose-based friction film was performed by a two-electrode system. The prepared friction film (size: 10 mm × 10 mm) is the intermediate layer of the capacitor. The electrolyte used was a LiCL solution of 1 mol L^−1^. The scan rate is set to 20–500 mV s^−1^, and the scan potential window is set to 0–1 V. The calculation Equation (1) of specific capacitance is as follows, and the calculation results are shown in Table 1:

(1) $$C=\frac{1}{2\cdot m\cdot s\cdot \Delta V}\int_{V_{0}}^{V_{0}+\Delta V}IdV$$

where m is the mass of the active substance on the electrode, s is the voltage scanning rate, ΔV is the potential drop in the whole cycle and *V*_0_ is the lowest voltage in the cycle. 

**Figure 3:**
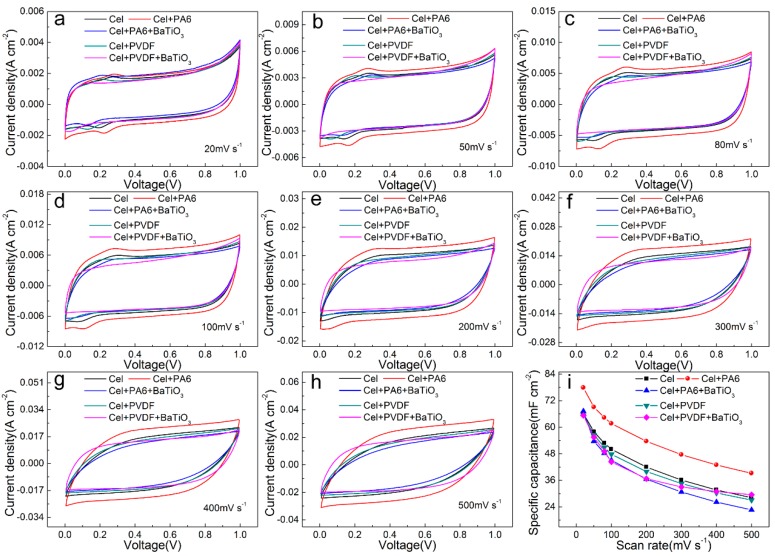
CV curve and specific capacitance curve of different types of friction layer: (**a**) 20 mV s^−1^, (**b**) 50 mV s^−1^, (**c**) 80 mV s^−1^, (**d**) 100 mV s^−1^, (**e**) 200 mV s^−1^, (**f**) 300 mV s^−1^, (**g**) 400 mV s^−1^, (**h**) 500 mV s^−1^, (**i**) specific capacitance.

Figure 3a–h are CV curves of different types of friction layers at a scan rate of 20–500 mV s^−1^. It can be seen from each CV curve that when the sweep speed is lower than 200 mV s^−1^, each friction layer has an obvious redox reaction peak. This is most obvious in the friction layer with the addition of PA6. After adding PVDF and BaTiO_3_, it has a certain inhibitory effect on the redox peak. I_pc_/I_pa_ ≈ 1 is obtained by calculation, which indicates that the whole cycle process is close to stable. However, due to the internal resistance of each friction layer, a small amount of leakage current is generated, which is slight deviation from the ideal state. At the same time, the smooth and non-virtual CV curve also shows that each friction layer has good electrochemical stability. As the sweep speed increases, the redox peak gradually shifts until it disappears, because the scan rate is too fast to react.

The corresponding specific capacitance value is obtained by calculating the area of the CV curve. Figure 3i and Table 1 show that the specific capacitance of the friction layer changes greatly after the addition of PA6, PVDF and BaTiO_3_. Specifically, after adding PA6, the specific capacitance of the friction layer is greatly increased. At 20 mV s^−1^, the specific capacitance increased from 66.225 mF cm^−2^ to 77.896 mF cm^−2^, which is increase by 1.176 times. At a sweep speed of 500 mV s^−1^, the specific capacitance increased from 28.156 mF cm^−2^ to 39.212 mF cm^−2^, which is increase by 1.393 times. Due to the certain redox reaction and the internal ion migration rate of each friction layer, the specific capacitance cannot increase with the increase of the scanning rate as the sweep speed increases, and the capacitance value of each friction layer decreases. On this basis, the friction layer is greatly reduced in specific capacitance after the addition of BaTiO_3_. At 20 mV s^−1^ sweep speed, the specific capacitance is reduced from 77.896 mF cm^−2^ to 67.252 mF cm^−2^, which is reduced to 0.863 times. At 500 mV s^−1^ sweep speed, the specific capacitance decreased from 39.212 mF cm^−2^ to 22.654 mF cm^−2^, which is reduced to 0.578 times. After adding PVDF, the specific capacitance of the friction layer is reduced. At 20 mV s^−1^, the specific capacitance decreases from 66.225 mF cm^−2^ to 65.509 mF cm^−2^, which is reduced to 0.989 times. At a sweep speed of 500 mV s^−1^, the specific capacitance decreases from 39.212 mF cm^−2^ to 27.078 mF cm^−2^, which is reduced to 0.962 times. On this basis, after adding BaTiO_3_, the friction layer ratio is reduced at low sweep speed. At 20 mV s^−1^, the specific capacitance decreases from 66.225 mF cm^−2^ to 65.293 mF cm^−2^, which is reduced to 0.997 times. At 500 mV s^−1^, the specific capacitance increases from 27.078 mF cm^−2^ to 29.488 mF cm^−2^, which increases by 1.089 times. 

Since the friction layer specific capacitance is related to its internal charge capacity and ion permeability, after adding PA6, the internal area of the friction layer increases, and the charge capacity increases, which cause the specific capacitance rises. After adding PVDF and BaTiO_3_, the specific capacitance of PVDF and BaTiO_3_-based cellulose films are lower than that of cellulose composite films. On the one hand, PVDF can easily obtain electrons by friction and is used as a negative material. Although its piezoelectric performance can make it a dopant, it can improve the performance of friction generators. But because it is easy to get electrons, the specific capacitance will be lower than that of pure cellulose film. On the other hand, it is because during the electrochemical test, the sample film is not squeezed without generating a dipole moment and producing a piezoelectric effect, and then its specific capacitance is reduced.

### 3.3. EIS and GCD Test Analysis

**Figure 4:**
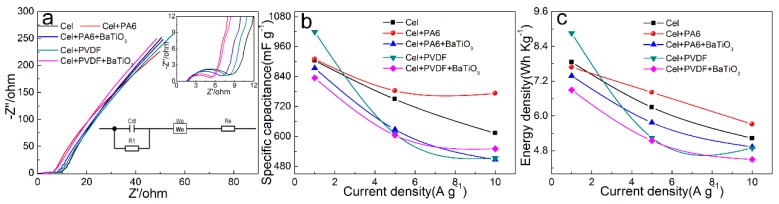
(**a**) EIS curve at 10^5^ Hz-10^−2^ Hz and GCD curves at different current densities: (**b**) specific capacitance, (**c**) energy density.

Electrochemical alternating current impedance spectroscopy (EIS) is an effective method for studying electrochemical processes. The AC impedance characteristics of cellulose-based friction film materials can reflect various kinds of information such as ion diffusion and charge transfer. The equivalent circuit parameters (Figure 4a) C_dl_ and ionic conductivity *σ* are calculated by Equations (2) and (3), as follows:

(2) $$C_{\mathrm{dl}}=\frac{1}{\omega \cdot R_{\mathrm{ct}}}$$

(3) $$\sigma =\frac{L}{R_{\mathrm{ct}}\cdot A}$$

where *ω* is the highest angular frequency of the arc, *L* is the thickness of the sample, and *A* is the surface area of the ionic electrolyte film.

The EIS curves of different types of friction layers are conducted at 10^5^ Hz–10^−2^ Hz (Figure 4a). The EIS graph curve is roughly divided into three sections: high, medium and low. The three sections are associated with the parameters of Re, W_0_, R_1_ and C_dl_ in the equivalent circuit. The semi-arc of the high frequency band is remarkable, indicating that each friction layer has stable and good electrochemical characteristics, and the arcs are different in size due to different materials. The equivalent resistance R_e_ is obtained by the intersection of the high-frequency arc area and the left side of the real axis, and R_e_ reflects the overall internal resistance of the friction layer. When other conditions are constant, the thickness of the friction layer increases and R_e_ increases after adding PA6, PVDF and BaTiO_3_. The mid-band is ideally a small 45° line segment, indicating the diffusion of ions into the pore structure of the friction layer. The slope changes due to the Warburg impedance region. It can be seen from the difference of the slope of the EIS curve that the difficulty of charge transfer in each friction layer system is different, and the slope of the EIS curve of the Cel friction layer is small, indicating that it has a large impedance charge transfer capability. The slope of the EIS curve of the friction layers after adding PA6, PVDF and BaTiO_3_ increased, which indicates that it has a small impedance value. W_0_ combines the high frequency and low frequency to obtain the overall transmission impedance value R_ct_. The charge transfer resistance R_ct_ reflects the difficulty of transferring charge (electrons and ions) into the friction layer. The size can be obtained from the high frequency band. The difficulty of transfer is inversely related to the size. The small diameter can cause a small transfer resistance, and there is a certain relationship with the frequency band in the test segment. The ideal low frequency band is a straight line parallel to the vertical axis, but due to the continuous current impact, there will be a certain leakage resistance R_1_ in the driver. Because it is small, it is usually ignored. The slope of the overall curve can reflect the speed of the ion diffusion rate of the whole process, and the conductivity σ can be obtained by formula calculation. 

The electric double layer capacitor C_dl_ reflects the internal charge capacity. As can be seen from Table 2, the value increases after adding PA6 and PVDF. After the addition of BaTiO_3_, the C_dl_ value further increases. The ionic conductivity σ is calculated by Equation (2). Combined with Table 2, it can be found that the σ value increases after adding PA6, PVDF and BaTiO_3_. After adding PA6, PVDF and BaTiO_3_, the increase of C_dl_ and σ indicates that the charge capacity and the charge transfer rate of the cellulose layer increase. Based on strategies to develop efficient TENG through increasing dielectric constant, optimization of internal resistance, and surface modification, the internal resistance of the cellulose friction film changes, and the electrical performance of the friction generator will be improved [34]. Thus it is estimated that the electrical output performance of TENG after assembly also increases.

Specific capacitance *C* and energy density *E* can be obtained by using Equations (4) and (5), where *m* is the mass of active substances on the electrode, *I* is the charging current, ΔV is the charging potential difference, and Δt is the charging time:

(4) $$C=\frac{I\cdot \Delta t}{m\cdot \Delta V}$$

(5) $$E=\frac{1}{2}\cdot C\cdot {\left(\Delta V\right)}^{2}$$

GCD curves of different types of friction layers at different current densities (1 A g^−1^, 5 A g^−1^, 10 A g^−1^, see Figure 2). Due to the internal resistance of each friction layer, the voltage drop at different current densities is obtained by processing the GCD curve (Figure 2). It can be seen that the voltage drop increases with the increase of current density. Among them, at low current density (<5 A g^−1^), the voltage drop of the friction layer all increases after adding PA6, PVDF and BaTiO_3_. The specific capacitance *C* is calculated by Equation (4), and the result is plotted as Figure 4b. It can be seen that since the ion migration transmission rate cannot be consistent with the current density increase amplitude. As the current density increases, the frictional capacitance of each friction layer first decreases sharply and then tends to be stable. In addition, after adding PA6, PVDF, and BaTiO_3_, the internal arrangement structure of each friction layer is different and lead to different permeability, so the electrochemical parameters are different at each current density. The energy density *E* is calculated according to Equation (5), and the result is plotted as Figure 4c. It can be found that the energy density has a similar change trend with the specific capacitance. Since this experiment uses a potential-based charge and discharge test method. By simplifying the calculation formula, it is known that the current density is the larger than the energy density, so it tends to decrease as the current density increases. Among them, at the current density of 1 A g^−1^, after adding PVDF, the energy density reaches 8.85 Wh Kg^−1^ (up to 112.78% of the pure cellulose friction layer). It can be found that the specific capacitance and energy density decrease after adding PA6, PVDF and BaTiO_3_. Although the number of internal conductive particles increases, but the permeability decreases, and the ion rate of transmission also decreases at low current densities. It is manifested that the specific capacitance and energy density are reduced at this current density.

### 3.4. Single Open Circuit Voltage/Short Circuit Current Test

**Figure 5:**
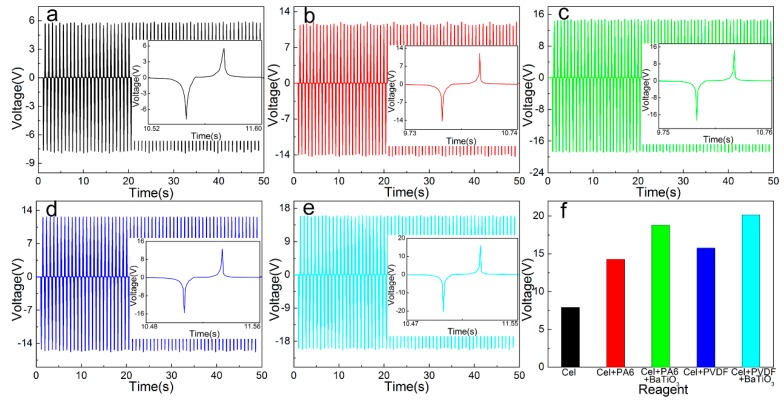
Output voltage of a single different type of friction layer: (**a**) Cel, (**b**) Cel + PA6, (**c**) Cel + PA6 + BaTiO_3_, (**d**) Cel + PVDF, (**e**) Cel + PVDF + BaTiO_3_, (**f**) maximum instantaneous value.

**Figure 6:**
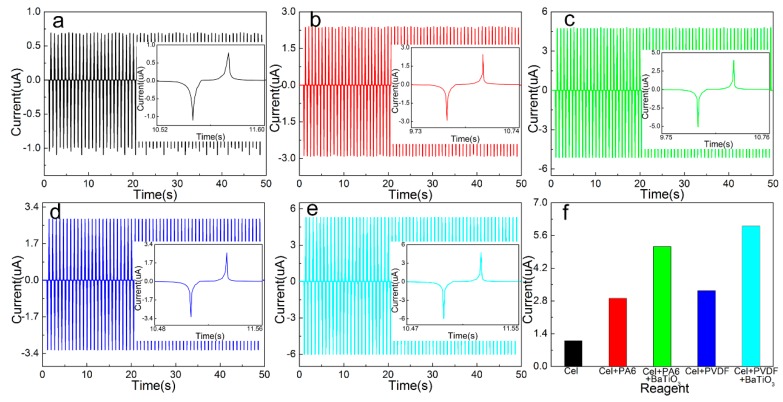
Output current of a single different type of friction layer: (**a**) Cel, (**b**) Cel + PA6, (**c**) Cel + PA6 + BaTiO_3_, (**d**) Cel + PVDF, (**e**) Cel + PVDF + BaTiO_3_, (**f**) maximum instantaneous value.

The performance of different types of friction layer and PTFE film is tested, and the extrusion frequency was 1 Hz. The open circuit output voltage and short circuit current values of different types of friction layers in the 50 s are tested experimentally. By eliminating the cluttered data and fitting the curve, the open circuit output voltage curve of a single different type of friction layer is shown in Figure 5, and the short circuit output current curve is shown in Figure 6. It can be seen that the peak output voltage of the pure cellulose friction layer is 7.925 V, and the peak value of the short-circuit output current is 1.095 μA. After adding PA6, the output voltage increased to 14.279 V (up 180.17%) and the output current increased to 2.917 μA (up 266.30%). After adding PVDF, the output voltage increased to 15.755 V (up 198.80%) and the output current increased to 3.239 μA (up 295.63%). Due to the different electronegativity of different materials, the electrical output performance is different. The greater different in electronegativity between the two materials, the better the electrical output performance of the nano-generator. Therefore, the electrical output performance after PVDF is better than that with PA6. After adding BaTiO_3_ based on the addition of PA6, the output voltage was further increased to 18.798 V (up 131.65%), and the output current was increased to 5.129 μA (up 192.59%). After adding BaTiO_3_ to the PVDF, the output voltage is further increased to 20.155 V (up 127.93%), and the output current is increased to 6.001 μA (up 185.30%). This is because BaTiO_3_ is a ferroelectric compound with high dielectric constant and low dielectric loss, and has a strong charge storage capacity. 

By using its high dielectric constant property, strong charge storage capacity and piezoelectric effect, its incorporation into the original friction layer can increase its dielectric constant and further improve the output performance of the nano-generator. At the same time, since the BaTiO_3_ crystal has no symmetry center, the charge distribution changes when the pressure is applied, the dipole moment is generated, and the piezoelectric effect is generated. The piezoelectric effect further enhances the piezoelectric performance of the friction layer and can also improve the output performance of the generator.

### 3.5. Multiple Combined Open Circuit Voltage/Short Circuit Current Test

**Figure 7:**
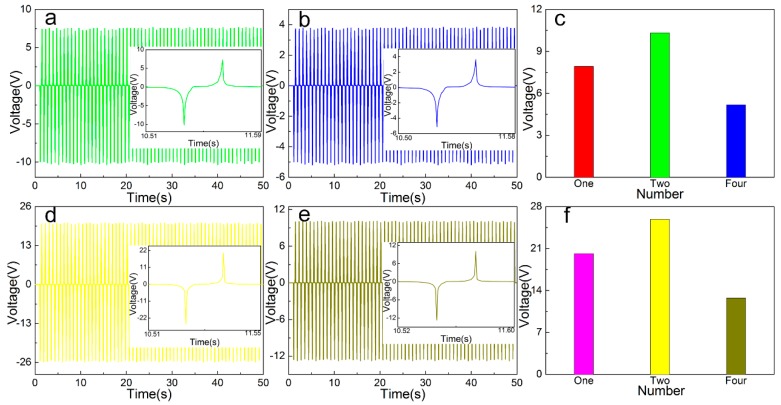
Output voltage of different number of TENG: (**a**) Cel, (**b**) Cel + PA6, (**c**) Cel + PA6 + BaTiO_3_, (**d**) Cel + PVDF, (**e**) Cel + PVDF + BaTiO_3_, (**f**) maximum instantaneous value.

**Figure 8:**
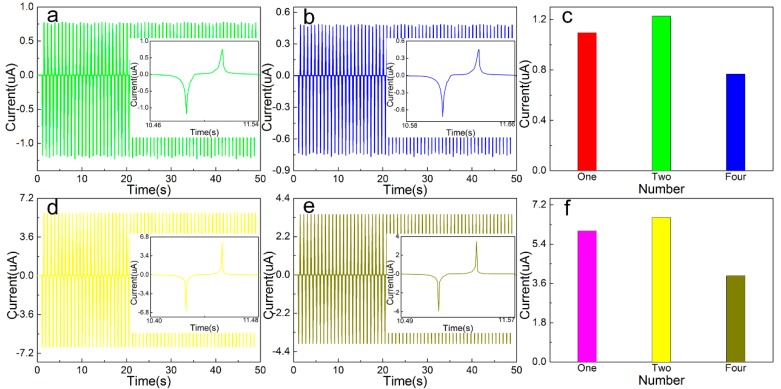
Output current of different number of TENG: (**a**) Cel, (**b**) Cel + PA6, (**c**) Cel + PA6 + BaTiO_3_, (**d**) Cel + PVDF, (**e**) Cel + PVDF + BaTiO_3_, (**f**) maximum instantaneous value.

For the TENG composed of different types of cellulose-based friction layers, different numbers are assembled, and the electrical output performance is tested after rectification and compared with a single TENG output parameter. The open-circuit output voltage and short-circuit current value of the friction layer TENG based on Cel, Cel + PVDF + BaTiO_3_ in 50 s are tested. The cluttered data is eliminated and the curve is fitted. The output voltage curves of different combinations are shown in Figure 7, short circuit output current curve is shown in Figure 8. It can be seen that the peak output voltage of the pure cellulose friction layer is 10.329 V (up 130.34%) and the peak value of the short-circuit output current is 1.227 μA (up 112.04%) compared to the single TENG output parameter. The output voltage of the friction layer based on Cel + PVDF + BaTiO_3_ is 25.872 V (up 128.37%), and the peak output current is 6.623 μA (110.37% increase). 

According to the experimental results, compared with a single TENG, the open circuit output voltage is increased by about 1.273–1.321 times after assembly, and the short-circuit output current peak is increased by about 1.110 times. This is due to the large friction layer resistance and the device’s own loss in rectifying process, resulting in the two after assembly open circuit output voltage and short circuit output current increase is small. After four assemblies, the output voltage of the open circuit based on the pure cellulose friction layer is 5.185 V (up 49.80% increase), and the peak value of the short-circuit output current is 0.767 μA (37.51% reduction). The output voltage of the friction layer based on Cel + PVDF + BaTiO_3_ is 12.755 V (50.71% reduction), and the peak output current is 3.947 μA (40.42% reduction). According to the experimental results, compared with a single TENG, the open circuit output voltage is reduced by 0.498–0.507 time, and the short-circuit output current peak is reduced by 0.375–0.405 time. After four assemblies, the open circuit output voltage and the short-circuit output current are greatly reduced mainly due to two aspects, namely, the power loss of the rectifying device and the asynchronous power of each TENG output. Subsequently, improving the output performance of the generator starts from reducing the power consumption of the rectifier bridge and ensures the phase synchronization of the integrated TENG output, which reduces the internal resistance of the friction layer.

### 3.6. Charge and Discharge Test

**Figure 9:**
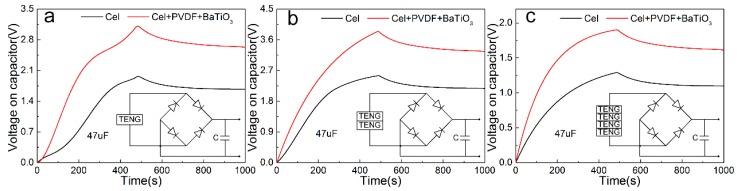
Charging and discharging performance test of different numbers of friction layers: (**a**) single, (**b**) two (**c**) four.

Different number of cellulose based nano-generators is used to supply power to the 47 μF capacitor, and the charge and discharge performance in 1000 s was tested. The first 500 s is used for power supply, and the power-down characteristics are observed after 500 s to facilitate the design of the subsequent energy storage circuit. Figure 9a,b,c are based on the Cel, Cel + PVDF + BaTiO_3_ friction layer, assembled single, two, four TENG charge and discharge patterns. It can be seen that the charging peak of a single Cel-based friction layer generator in the 500 s reaches 1.965 V, and the power is reduced to 1.672 V, and the power failure rate is 14.91%. The charging peak value of the Cel + PVDF + BaTiO_3_ friction layer generator reaches 3.114 V, and the power is reduced to 2.628 V, and the power failure rate is 15.61%. The charging peak of two Cel-based friction layer generators reaches 2.554 V, and the power is reduced to 2.177 V, and the power-down rate is 14.76%. The charging peak value of the Cel + PVDF + BaTiO_3_ friction layer generator reaches 3.840 V, and the power is reduced to 3.254 V, and the power failure rate is 15.26%. The charging peak of four Cel-based friction layer generators reaches 1.291 V, and the power is reduced to 1.098 V, and the power-down rate is 14.69 %. The charging peak value of the Cel + PVDF + BaTiO_3_ friction layer generator reaches 1.903 V, and the power is reduced to 1.612 V, and the power failure rate is 15.18%. According to the test results, compared with the single TENG charge and discharge, the two TENG charging rates and peaks are improved at the same time. The charging peaks based on the Cel, Cel + PVDF + BaTiO_3_ friction layer increases to 129.96% and 123.33%, respectively. Four TENG charging rates and peak values are significantly reduced by 49.46% and 50.45%, respectively. This corresponds to the open circuit output voltage after assembly in the previous test. In addition, for TENG consisting of the same friction layer, the power-down rate of TENG after twice and four times charge is basically the same. Among them, the combined power-down rate has decreased, indicating the stability of multiple combined TENG. The offset rate also ensures subsequent powering of the MCS-52 microcontroller, which in turn drives the SFM-27 buzzer alarm and illuminates the 1602 LCD.

## 4. Conclusions

In summary, this paper proposes an environmentally-friendly triboelectric- piezoelectric nano-generator based on degradable cellulose, which utilizes cellulose that can be extracted from many natural vegetation sources and organisms. By utilizing the characteristics of cellulose-IL dissolution regeneration, regenerated cellulose film is obtained as a friction and piezo-layer. Then, it is assembled with a PTFE film to form TENG that generates electricity by extrusion friction, and tested for electrical output properties. The results show that the peak value of open circuit output voltage increases by 2.544 times to 20.155 V, and the peak value of short circuit output current increases by 5.480 times to 6.001 μA. After two assemblies, the open-circuit output voltage is increased to a maximum value by 1.321 times, and the short-circuit output current peak is increased to a maximum value by 1.161 times. After four assemblies, the open circuit output voltage and short circuit output current are greatly reduced. This is due to the large internal resistance of the friction layer and the loss of the device itself during the rectification process and the phase frequency of the TENG output are not synchronized, resulting in a small increase in the output voltage and short-circuit output current after assembly. Within 500 s, the charge peak of single charge, two reaches and four charge reaches 3.114 V, 3.840 V, and 1.903 V, respectively. This ensures long-term storage of electrical energy and subsequent supply of power to the electronics. The test shows that the proposed cellulose-based nano-generator device has good stability and reliability, and can be widely used in the field of energy harvesting conversion storage, which greatly promotes the application and development of natural biomaterials in the field of TENG and other electronic sensing devices.

## Figures and Tables

**Table 1:** Specific capacitance values of different friction layers at different scanning rates.

	Reagent	Cel	Cel + PA6	Cel + PA6 + BaTiO_3_	Cel + PVDF	Cel + PVDF + BaTiO_3_
Scan Rate (mv s^−1^)						
20		66.225	77.896	67.252	65.509	65.293
50		57.928	69.077	53.678	56.498	55.491
80		52.807	64.34	48.245	50.942	49.077
100		50.057	61.642	45.053	47.768	44.318
200		42.004	53.631	36.545	39.974	36.716
300		36.112	47.611	30.721	34.529	32.986
400		31.625	42.921	26.148	30.301	30.953
500		28.156	39.212	22.654	27.078	29.488

**Table 2:** Parameters of different types of friction layer.

	Reagent	Cel	Cel + PA6	Cel + PA6 + BaTiO_3_	Cel + PVDF	Cel + PVDF + BaTiO_3_
EIS Parameter						
R_e_ (Ω)		1.455	1.553	1.571	1.922	1.731
R_ct_ (Ω)		7.194	3.876	5.986	6.794	4.324
C_dl_ (mF)		0.439	0.453	0.572	0.504	0.792
σ (ms/cm)		3.029	10.423	7.947	8.613	14.738
